# Supplementary figures and images for: Rule-based habitat suitability modelling for the reintroduction of the grey wolf (Canis lupus) in Scotland
Source: PLoS One. 2022 Oct 21;17(10):e0265293. doi: 10.1371/journal.pone.0265293 (PMC9586354; doi:10.1371/journal.pone.0265293)

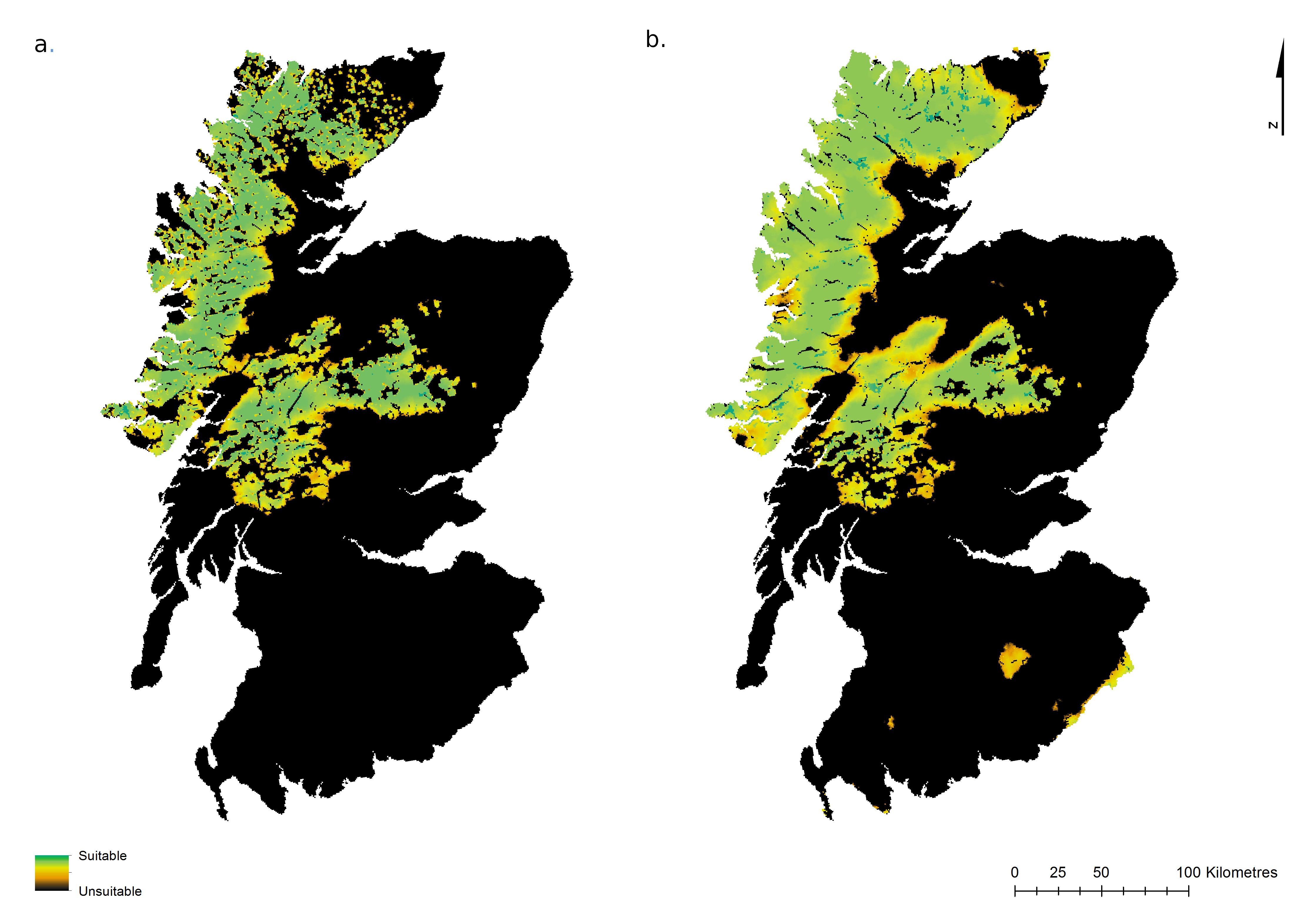

Supplement: S1 Fig — These were produced by Model 2 (a) and Model 5 (b) in Table 4, respectively. (TIFF) [file pone.0265293.s001.tiff]
